# Meta-Accuracy on the Internet: Initial Tests of Underlying Dimensions, Contributing Factors, and Biases

**DOI:** 10.3389/fpsyg.2022.837931

**Published:** 2022-03-02

**Authors:** Elena Tsankova, Ergyul Tair

**Affiliations:** Personality Psychology and Methodology of Psychological Measurement, Department of Psychology, Institute for Population and Human Studies, Bulgarian Academy of Sciences, Sofia, Bulgaria

**Keywords:** meta-accuracy, first impressions, Internet, warmth, competence, emotional intelligence, expressivity, bias

## Abstract

Meta-accuracy (correspondence between how we think others perceive us and how they really perceive us) of first impressions on the Internet has the potential to shape subsequent interactions. Aiming to enhance understanding of the underlying perceptual dimensions, the contribution of social competence, and the existence of positive/negative bias in first impressions’ meta-accuracy online, we conducted a study in a simulated asynchronous social-media-type setting. Target participants uploaded a selfie, wrote a short description of themselves, provided estimates of how warm and competent they believed others would find them based on their selfies and texts (metaperception), and completed two social competence questionnaires (general and Internet-specific). Perceiver participants assessed the warmth and competence of the selfies and texts as well (others’ perception). Meta-accuracy was measured as the absolute difference between metaperception and others’ perception. Through correlational analyses, we confirmed that meta-accuracy of first impressions on the Internet aligned with the universal dimensions of social cognition (warmth and competence), found sporadic evidence for the positive association between meta-accuracy and social competence, and showed that meta-accuracy for specific Internet expressive means varied with varying proficiency in these means. Through *t*-tests, we demonstrated positive meta-accuracy bias for selfies along the warmth dimension and negative bias for text along the competence dimension. Overall, our results suggest the primacy of warmth and uniqueness of the male targets-female perceivers combination for meta-accuracy on the Internet. Our findings expand knowledge about first impressions’ meta-accuracy on the Internet.

## Introduction

### First Impressions, Accuracy, and Meta-Accuracy

First impressions (inferences about others’ personalities and intentions) occur instantaneously (e.g., Bar et al., 2006; Willis and Todorov, 2006). They can be remarkably durable (e.g., Gunaydin et al., 2017) and influential (e.g., Harris and Garris, 2008; Olivola and Todorov, 2010). From an evolutionary perspective, people who form accurate first impressions have an advantage, as they can quickly and correctly infer others’ intentions and choose appropriate behavioral responses such as approach or avoidance (e.g., Zebrowitz and Montepare, 2006). Equally important for choosing the best reaction for survival is to accurately understand how others perceive us. The accuracy of such inferences entails a metacognitive component (i.e., thinking about what others are thinking) and has thus been termed “meta-accuracy” (Kenny and DePaulo, 1993). The meta-accuracy of first impressions may determine subsequent interaction by affecting both attitudes and behavior. For example, incorrectly inferring that someone has formed a negative first impression about us may cause us to avoid them. Hence, in the study of first impressions, it is essential to also address their meta-accuracy.

### First Impressions’ Meta-Accuracy on the Internet

First impressions occur in all types of first encounters, including Internet communication. The study of meta-accuracy of Internet-based first impressions, however, is still in its early days. To the best of our knowledge, there are only three reports explicitly dedicated to the meta-accuracy of Internet-type first impressions (self-favoring bias in selfie perception, Re et al., 2016; personality expression and impression formation in online social networks, Stopfer et al., 2014; impression management in online social network sites, Wu and Zheng, 2019). First impressions’ meta-accuracy has largely been studied in face-to-face interaction (Tsankova and Tair, 2021a), but it is likely that certain specifics of Internet communication, such as expressive means (e.g., selfies favoring readily inferable static cues, videos, and teleconferencing favoring less readily inferable dynamic cues, written text, emoji, etc.) and synchronicity (e.g., the amount of time available for inference making and response) may also affect it. Given the ever-increasing significance of Internet communication, especially in the context of the COVID-19 pandemic, it is crucial to achieve a proper understanding of meta-accuracy of first impressions on the Internet. Enhancing knowledge on the matter would add to both theoretical understanding of and improving Internet communication on the practical level (e.g., help with avoiding misinterpreting intentions on dating websites).

### Factors Contributing to First Impressions’ Meta-Accuracy

Meta-accuracy depends on various factors classified into four categories: information, traits, targets, and judges (Funder, 1995; Carlson and Elsaadawy, 2021). In this study, we refer to judges as perceivers as we wish to emphasize the spontaneous rather than deliberative nature of first impressions.

Recent evidence of impaired first impressions’ meta-accuracy in autism (Sasson et al., 2018) suggests that certain aspects of social competence related to the impression target may be associated with meta-accuracy. To examine this link further, but as social competence entails many components, which vary depending on the applied theoretical model, we applied an intuitive bottom-up approach in selecting the ones for our research. To cover both general and Internet-specific aspects of social competence, we opted for studying meta-accuracy about emotional intelligence and Internet expressivity proficiency.

### Underlying Dimensions of First Impressions’ Meta-Accuracy

Finally, in the study of first impressions, it is important to determine what dimensions they would cover. First impressions of unfamiliar people typically consist of cognitive and affective categorizations on two dimensions of social cognition, namely, warmth and competence (Fiske et al., 2007). It is reasonable to assume that the metacognitive aspect of first impressions would follow the same dimensions. Initial evidence supporting this claim comes from a series of experiments on the metaperception of morality (Rom and Conway, 2018). Here, to provide further support for this finding and extend it to online settings, we chose warmth and competence as dimensions for studying first impressions’ meta-accuracy on the Internet.

### Aim and Hypotheses

In this study, we sought to take a step further in the study of first impressions’ meta-accuracy on the Internet by examining its underlying perceptual dimensions and contributing factors. In particular, we wished to check whether meta-accuracy of first impressions on the Internet followed the universal dimensions of social cognition and whether it was affected by a target-specific factor, namely, social competence. We made the following predictions:

#### Hypothesis 1 (H1)

Meta-accuracy of first impressions on the Internet would align with and be measurable along the universal dimensions of social cognition, and this alignment would be revealed by positive correlations between targets’ metaperceptions and perceivers’ impressions of the targets along these dimensions.

#### Hypothesis 2 (H2)

Meta-accuracy of first impressions on the Internet would increase with increasing general and Internet-specific social competence.

#### Hypothesis 3 (H3)

Meta-accuracy of first impressions on the Internet would depend on proficiency with Internet-specific expressive means in such a way that higher proficiency with a particular means would be associated with higher meta-accuracy for this means.

On an exploratory basis, we also checked for sex, social dimension, social competence, and expressive mean differences, as well as for positive/negative bias in meta-accuracy of first impressions on the Internet.

## Materials and Methods

### Preregistration

The study was registered on the Open Science Framework prior to data collection (protocol).^1^ Further, *a priori* power analyses in G*Power (Version 3.1.9.4, Faul et al., 2007, 2009) are available in Supplementary Material (p. 1). A slight deviation from preregistration was the increased number of perceivers intended to enhance average rating robustness. We also omitted the outlier analysis, as we wished to reflect reality as closely as possible, and accepted that a couple of extreme values may be present in a standard sample. However, we performed normality checks and used the appropriate non-parametric tests when normality was violated. Comparing parametric and non-parametric tests did not reveal differences in results. Finally, according to the preregistration protocol, Hypothesis 3 was initially tested with repeated measures analyses (RM-ANOVAs), and the results are available in Supplementary Material (p. 8). Following a reviewer’s suggestion, however, we eventually favored an approach that considers the continuous nature of the variables. Both analyses showed similar results.

### Setting

To avoid procedural and ethical pitfalls associated with the study of meta-accuracy on the Internet (Tsankova and Tair, 2021b), we opted for a simulated asynchronous setting of first encounters online.

### Operationalization of Social Competence

We measured general social competence with an emotional intelligence questionnaire (the Trait Emotional Intelligence Questionnaire-Short Form, or TEIQue-SF; Petrides, 2009) and Internet-specific social competence in terms of Internet expressivity (the Internet Expressivity Proficiency Questionnaire, or IEPQ, created especially for this study, Supplementary Material, pp. 4–5).

### Targets

#### Participants

We selected targets *via* Prolific.^2^ Entry requirements were minimum and maximum age (18 and 35 years, respectively) and fluent command of the survey language (English). From the 217 (106 females) participants who entered the survey, 189 (93 females, *M*_age_ = 23.39, *SD*_age_ = 3.99, age range = 18–35 years) participants provided valid data and were included as targets. Information on data screening and exclusion is provided in Supplementary Material (p. 2).

#### Materials

Materials consisted of the two social competence questionnaires (TEIQue-SF and IEPQ), the warmth and competence judgment questions (7-point Likert-type scales, 1 = *not at all*, 7 = *completely*), and three control questions (scales as above, Supplementary Material, p. 10). Warmth and competence definitions were based on Fiske et al. (2007) and are provided in Supplementary Material (p. 3).

#### Procedure

The study was programmed and conducted using the SoSci Survey.^3^ Participants uploaded a selfie and wrote a brief text describing their personality (2–3 sentences or 100–600 characters in length, including spaces). They were instructed to imagine that their content could be used on social media serving both personal and professional purposes. Next, participants provided estimates of how warm and how competent others might perceive them to be based on their selfies and texts (META ratings). General self-evaluation ratings along the two dimensions, as well as self-evaluation of their selfies and texts along the dimensions were also obtained but are omitted here as they are not relevant to this report. Finally, participants answered the control questions and completed the social competence measures (internal consistency is reported in Table 1).

**TABLE 1:** Observed internal consistency for the social competence measures.

		Number of items	Cronbach’s α estimate	Cronbach’s α 95% CI (LL, UL)
TEIQue-SF	Well-being	6	0.84	0.80, 0.87
	Self-control	6	0.70	0.63, 0.76
	Emotionality	8	0.74	0.67, 0.79
	Sociability	6	0.74	0.68, 0.79
	Global trait EI	30	0.91	0.89, 0.93
IEPQ-self	General	5	0.70	0.62, 0.76
	Feelings	5	0.81	0.76, 0.85
	Thoughts	5	0.79	0.73, 0.83
	Total	15	0.91	0.89, 0.93
IEPQ-meta	General	5	0.82	0.77, 0.86
	Feelings	5	0.84	0.80, 0.87
	Thoughts	5	0.83	0.78, 0.86
	Total	15	0.94	0.92, 0.95

### Perceivers

#### Participants

Perceivers were also selected *via* Prolific with entry requirements identical to the targets. Through Prolific prescreening, we confirmed that perceivers had not previously taken part as targets. From the 389 (196 females) participants who entered the survey, 382 (192 females, *M*_age_ = 24.37, *SD*_age_ = 4.95, age-range = 18–35 years) participants provided valid data and were included as perceivers (information on data exclusion is given in Supplementary Material, p. 2).

#### Materials

Stimuli were the targets’ selfies and texts. For ecological validity, stimuli were mostly presented as provided (minor exceptions are given in Supplementary Material, p. 2). The warmth and competence evaluation questions followed the targets’ format.

#### Procedure

The perceiver survey was also programmed and conducted using the SoSci Survey. Perceivers evaluated the target stimuli along the warmth and competence dimensions (OTHER ratings). Stimuli were grouped in clusters based on the order in which target data had arrived. Stimuli were randomized within clusters. Each cluster was judged by 18–21 perceivers (perceiver sex was counterbalanced as much as possible). The exact distribution of targets and perceivers per cluster is available in Supplementary Material (p. 7).

## Results

### Meta-Accuracy Index

For our purposes, meta-accuracy was the correspondence between targets’ metaperception and perceivers’ impressions of the targets. To obtain a numerical value for this correspondence, we subtracted, for each target and combination of social dimension and expressive means, the averaged OTHER ratings from all perceivers from the respective target’s META rating. To use the resulting values, which could be both positive and negative, without interfering with the planned tests’ computation, we took the absolute value of the difference.

Notably, a larger meta-accuracy index indicates a larger difference between meta-and-other perceptions and, thus, a lower meta-accuracy. Therefore, by correlating the meta-accuracy index with social competence, we examined negative correlations (with increasing social competence, the meta-accuracy index should decrease and meta-accuracy should increase).

Social competence scores (according to each tool’s instructions) and meta-accuracy indices were computed using Microsoft Excel, while statistical analyses were conducted using JASP (Version 0.14.1.0, JASP Team, 2021).

### Results

We reported our results on the level of the entire sample as well as on the level of the smallest possible sample split, which was the four target-perceiver sex combinations.

#### Hypothesis 1 (H1)

To test whether meta-accuracy of first impressions on the Internet was measurable along the universal dimensions of social cognition, we computed a series of one-tailed positive Pearson correlations between our targets’ META ratings and our perceivers’ averaged OTHER ratings for all combinations of dimension, expressive means, and, on an exploratory basis, target-perceiver sex (Table 2). As expected, targets’ metaperception was positively and significantly correlated with perceivers’ impressions (most *p*-values were < 0.05), suggesting that metaperception for both types of Internet-specific expressive means that we studied occurred along the universal dimensions of social cognition. Notably, the effect was larger for warmth (medium to large, *r* ranging from 0.23 to 0.61) than for competence (small to medium, *r* ranging from 0.20 to 0.31).

**TABLE 2:** Pearson correlations between targets’ metaperceptions (META ratings) and perceivers’ average impressions (average *other* ratings).

	Targets’ metaperception									

	Selfie					Text				
		
	All	FF	FM	MF	MM	All	FF	FM	MF	MM
	**Warmth**									
Perceivers’ average impressions	0.46***	0.41***	0.44***	0.41***	0.42***	0.49***	0.23*	0.28**	0.61***	0.46***
	**Competence**									
Perceivers’ average impressions	0.23***	0.31**	0.23*	0.11	0.20*	0.23***	0.25*	0.27**	0.23*	0.11

*All, all targets, all perceivers; FF, female targets, female perceivers; FM, female targets, male perceivers; MF, male targets, female perceivers; MM, male targets, male perceivers. N_All_, 189; n_FF_, 93; n_FM_, 93; n_MF_, 96; n_MM_, 96.*

*p < 0.05, **p < 0.01, ***p < 0.001, one-tailed for positive correlation.

#### Hypothesis 2 (H2)

We checked whether social competence was positively associated with meta-accuracy in a series of one-tailed Pearson correlations for each combination of measure, dimension, expressive means, and, on an exploratory basis, target-perceiver sex (Table 3). Contrary to our expectations, social competence was mostly uncorrelated with meta-accuracy.

**TABLE 3:** Pearson correlations between meta-accuracy index and social competency measures.

		Meta-accuracy index									

		Selfie					Text				
			
		All	FF	FM	MF	MM	All	FF	FM	MF	MM
		**Warmth**									
TEIQue-SF	Well-being	0.13	0.11	0.12	0.05	0.15	0.04	0.07	0.13	−0.09	0.03
	Self-control	0.22	0.14	0.16	0.21	0.26	−0.01	0.03	0.05	−0.07	−0.05
	Emotionality	0.10	−0.05	−0.09	0.21	0.23	−0.04	0.05	0.01	−0.17^†^	−0.05
	Sociability	0.14	0.10	0.18	0.06	0.17	−0.02	0.03	0.06	−0.19*	0
	Global trait EI	0.18	0.11	0.13	0.16	0.24	0.01	0.07	0.09	−0.13	−0.02
IEPQ-self	General	0.07	−0.04	0.09	0.09	0.10	−0.07	−0.13	−0.07	−0.09	0.02
	Feelings	0.13	0.01	0.12	0.16	0.16	−0.11	−0.18*	−0.10	−0.11	−0.01
	Thoughts	0.09	−0.03	0.08	0.14	0.14	−0.06	−0.02	−0.07	−0.09	−0.03
	Total	0.11	−0.02	0.10	0.14	0.14	−0.09	−0.12	−0.09	−0.10	−0.01
IEPQ-meta	General	0.15	0.09	0.18	0.14	0.14	−0.10	−0.05	−0.04	−0.23*	−0.05
	Feelings	0.12	0.06	0.08	0.15	0.13	−0.05	0.03	−0.01	−0.15	−0.04
	Thoughts	0.07	−0.03	0.08	0.08	0.10	−0.08	−0.01	−0.06	−0.17^†^	−0.08
	Total	0.12	0.04	0.12	0.13	0.13	−0.08	−0.01	−0.04	−0.19*	−0.06
		**Competence**									
TEIQue-SF	Well-being	−0.12^†^	0.03	−0.02	−0.28**	−0.12	0	−0.04	0.09	−0.05	0.06
	Self-control	−0.10	−0.09	−0.10	−0.16^†^	−0.07	0.02	0.07	0.13	−0.13	0.04
	Emotionality	−0.05	0.17	0.05	−0.21*	−0.07	−0.01	−0.02	0.08	−0.02	0.02
	Sociability	−0.10	−0.10	−0.07	−0.15	−0.05	−0.05	−0.10	−0.04	−0.08	0.10
	Global trait EI	−0.12^†^	0.01	−0.04	−0.26**	−0.11	−0.02	−0.06	0.08	−0.08	0.05
IEPQ-self	General	0.03	0.01	0.17	0	−0.06	0.05	0.18	0.12	−0.01	−0.08
	Feelings	0.04	0.08	0.21	−0.02	−0.04	0.10	0.21	0.18	−0.02	−0.02
	Thoughts	0.07	0.05	0.19	0.01	0.01	0.12	0.27	0.20	−0.04	−0.02
	Total	0.05	0.05	0.22	0	−0.03	0.10	0.24	0.19	−0.03	−0.04
IEPQ-meta	General	0.03	0.05	0.15	−0.02	0	0.13	0.26	0.22	0	0.03
	Feelings	0	0.09	0.20	−0.08	−0.08	0.11	0.24	0.18	0.04	0.02
	Thoughts	0.02	0.05	0.19	−0.05	−0.03	0.08	0.18	0.13	0.01	0.01
	Total	0.02	0.07	0.19	−0.05	−0.04	0.11	0.24	0.19	0.02	0.02

*All, all targets, all perceivers; FF, female targets, female perceivers; FM, female targets, male perceivers; MF, male targets, female perceivers; MM, male targets, male perceivers. N_All_ = 189, n_FF_ = 93, n_FM_ = 93, n_MF_ = 96, n_MM_ = 96.*

*p < 0.05, **p < 0.01, ^†^0.05 ≤ p ≤ 0.07, one-tailed for negative correlation (higher meta-accuracy index signals larger difference between targets’ metaperception and others’ perception of them, thus indicating lower meta-accuracy).

We found the expected associations almost exclusively in the combination of male targets and female perceivers. In particular, along the warmth dimension, meta-accuracy for text was positively associated with TEIQue-SF sociability (*r* = −0.19, *p* = 0.031) and marginally positively associated with emotionality (*r* = −0.17, *p* = 0.053). Also, along the warmth dimension, meta-accuracy for text was positively associated with IEPQ metaperceptual proficiency in general (*r* = −0.23, *p* = 0.013) and overall Internet expression (*r* = −0.19, *p* = 0.031) and marginally positively associated with metaperceptual proficiency in expressing thoughts on the Internet (*r* = −0.17, *p* = 0.049). Along the competence dimension, meta-accuracy for selfies was positively linked to TEIQue-SF well-being (*r* = −0.28, *p* = 0.031), emotionality (*r* = −0.21, *p* = 0.018), and global emotional intelligence (*r* = −0.26, *p* = 0.005), with a tendency to be also positively linked to self-control (*r* = −0.16, *p* = 0.057). It is likely that these associations specific to the male targets-female perceivers case led to the marginal associations that appeared for all participants together (both well-being and global *r* = −0.12, *p* = 0.047). For the competence dimension, we did not find significant associations between meta-accuracy and Internet expressivity proficiency (*p* > 0.1).

Only for the combination of female targets and female perceivers did we find another significant association, and it was along the warmth dimension between text meta-accuracy and IEPQ self-perceived proficiency in expressing feelings on the Internet (*r* = −0.18, *p* = 0.041).

#### Hypothesis (H3)

We checked whether meta-accuracy for each type of expressive means varied with varying proficiency in the expressive means as measured by the respective IEPQ questions. Due to the inverse nature of the meta-accuracy index, we tested for one-tailed negative associations, which would translate to positive associations between meta-accuracy and IEPQ.

On the level of the sample (*n* = 198), there was only a tendency for selfie warmth meta-accuracy to be associated with IEPQ-self general proficiency in text-based Internet expression (*r* = −0.11, *p* = 0.068).

No significant associations emerged between meta-accuracy and Internet expression proficiency on the level of the female targets-female perceivers and the combinations of female targets-male perceivers (*n* = 93), and all the *p*-values were ≥ 0.1.

For the male targets-female perceivers pairing (*n* = 96), meta-accuracy for text warmth was associated with IEPQ-meta general proficiency in text-and-image-based Internet expression (*r*_text_ = −0.30, *p*_text_ = 0.002, *r*_image_ = −0.20, *p*_image_ = 0.028) and with IEPQ-meta text-based expression of thoughts (*r* = −0.21, *p* = 0.021). Meta-accuracy for text warmth also tended to be associated with IEPQ-self text-based expression of feelings (*r* = −0.16, *p* = 0.066), and selfie competence tended to be associated with IEPQ-meta text-based expression of feelings (*r* = −0.16, *p* = 0.059).

Finally, for the combination of male targets-male perceivers, meta-accuracy for selfie competence was associated with IEPQ-meta text-based expression of feelings (*r* = −0.20, *p* = 0.027).

Overall, the expected association between proficiency, in particular, Internet expressive means, and meta-accuracy for this means was present only in the case of text and almost exclusively within the male targets-female perceivers group.

#### Further Exploratory Analyses

##### Meta-Accuracy Biases

To further investigate the matter of potential metaperceptual biases, we conducted a series of two-tailed one-sample *t*-tests comparing the difference between meta-and-other perception with zero (inspired by Lu et al., 2018). A value of zero indicates perfect meta-accuracy, i.e., no difference between metaperception and others’ perception. A positive value for the difference score indicates a positive bias in metaperception, i.e., targets’ metaperception was more positive than others’ perception. Likewise, a negative value for the difference score indicates negative bias in metaperception with targets’ metaperception more negative than perceivers’ impressions. Thus, the *t*-tests allowed to check whether there was a significant deviation from perfect meta-accuracy (i.e., bias) and in the direction of the bias. The tests were conducted for the entire sample, as well as for each combination of dimension, expressive means, and target-perceiver sex.

On the sample level, we found significant deviations from perfect meta-accuracy in a positive direction for warmth, for both selfies [Student’s *t*(188) = 4.66, *p* < 0.001, *d* = 0.34, *M* = 0.46, *SD* = 1.35] and text [Student’s *t*(188) = 2.54, *p* = 0.012, *d* = 0.18, *M* = 0.23, *SD* = 1.26]. Along the competence dimension, a significant deviation was present only for text, and it went in a negative direction [Student’s *t*(188) = −2.86, *p* = 0.005, *d* = −0.21, *M* = −0.29, *SD* = 1.39).

**FIGURE 1:**
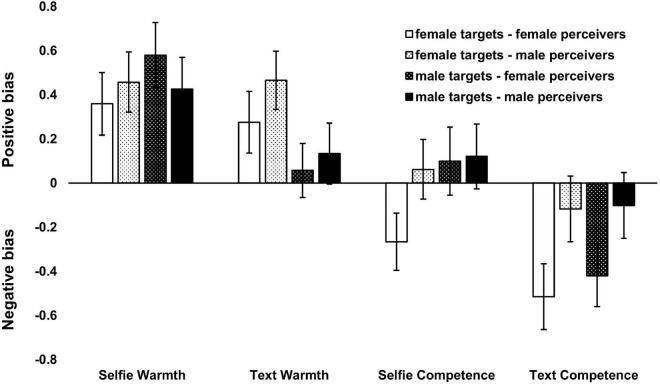
Metaperceptual biases. Results from the one-sample *t*-tests comparing the META, average OTHER difference with a perfect meta-accuracy value of zero for all combinations of dimension, expressive means, and target-perceiver sex. Error bars indicate ± 1 standard error of the mean (SEM).

On the level of the target-perceiver sex split (Figure 1), we found the most biases in the case of the combination of female targets-female perceivers. There, for warmth, a positive bias was present for selfies [Student’s *t*(92) = 2.53, *p* = 0.013, *d* = 0.26, *M* = 0.36, *SD* = 1.37], and a tendency for a positive bias was present for text [Student’s *t*(92) = 1.97, *p* = 0.052, *d* = 0.20, *M* = 0.28, *SD* = 1.35]. The least amount of bias was found in the combination of male targets-male perceivers, where it was present in a positive direction only for warmth and only for selfies [Student’s *t*(95) = 2.96, *p* = 0.004, *d* = 0.30, *M* = 0.43, *SD* = 1.41]. Positive bias along the warmth dimension was observed also in the female targets-male perceivers pairing, for both selfies [Student’s *t*(92) = 3.34, *p* = 0.001, *d* = 0.35, *M* = 0.46, *SD* = 1.32] and text [Student’s *t*(92) = 3.54, *p* < 0.001, *d* = 0.37, *M* = 0.47, *SD* = 1.27], as well as in the male targets-female perceivers pairing for selfies [Student’s *t*(95) = 3.95, *p* < 0.001, *d* = 0.40, *M* = 0.58, *SD* = 1.44]. For the combination of male targets-female perceivers, we also found a negative metaperceptual bias along the competence dimension for text [Student’s *t*(95) = −3.01, *p* = 0.003, *d* = −0.31, *M* = −0.42, *SD* = 1.37]. A negative competence bias was present also for the combination of female targets-female perceivers, for both selfies [Student’s *t*(92) = −2.05, *p* = 0.044, *d* = −0.21, *M* = −0.27, *SD* = 1.25] and text [Student’s *t*(92) = −3.46, *p* < 0.001, *d* = −0.36, *M* = −0.52, *SD* = 1.44].

To sum up, the warmth dimension was associated with a positive bias, mainly for selfies, but in some cases also for text. The competence dimension was associated with a negative bias mainly for text and in one case for selfies. The warmth bias appeared in more instances than the competence bias. Most biases were evident in the combination of female targets-female perceivers, while the least bias was observed in the combination of male targets-male perceivers.

##### Meta-Accuracy Differences

Information on meta-accuracy differences is provided in Supplementary Material (pp. 9–10).

##### Checks

Information on checks is provided in Supplementary Material (p. 10).

## Discussion

### Summary and Interpretation of Findings

In a simulated asynchronous online environment resembling first encounters on social media, we examined the underlying perceptual dimensions, the contribution of social competence, and the existence of positive/negative bias in meta-accuracy of first impressions on the Internet.

#### Underlying Dimensions

We showed that meta-accuracy of first impressions on the Internet followed the universal dimensions of social cognition, namely, warmth and competence. The effect was more pronounced for warmth, resembling Rom and Conway’s findings (2018). We attribute this asymmetry effect to the primacy of the warmth dimension (Fiske et al., 2007). As explained in Fiske et al. (2007), from an evolutionary point of view, people judge warmth before competence because it carries more information related to immediate survival (e.g., what others’ intentions may be). Thus, warmth estimates also contribute more strongly to affective and behavioral responses (e.g., approach–avoidance) than do competence estimates. From our findings, it appears likely that the primacy of warmth not only makes it faster and easier to process but also increases accuracy in terms of correspondence between metaperception and perception by others. In fact, our supplementary comparisons did indicate higher meta-accuracy for warmth. This finding adds to knowledge about similarities and differences between online and offline social cognition (e.g., Sparrow and Chatman, 2013, for review) by suggesting a correspondence between the two settings in terms of underlying perceptual dimensions guiding first impressions’ meta-accuracy.

#### Contributing Factors

Although our two social competence measures were positively, yet weakly, correlated with one another (Supplementary Checks), they were only sporadically correlated with first impressions’ meta-accuracy. On the one hand, it is plausible that social competence may simply not have been relevant in the experimental settings where no actual interaction occurred and where ecological validity might have been too low. On the other hand, it is likely that the measures do not address the precise aspects of social competence associated with meta-accuracy in our particular setting. The IEPQ is still under development and at present not distinguishing very reliably between self-and meta-perception.

Being able to imagine how unfamiliar others would perceive our online content requires successfully taking their perspective. However, the TEIQue-SF only contains one item addressing perspective-taking. We correlated meta-accuracy with responses to this item only (Supplementary Material, p. 11) and, in fact, found evidence for a positive association between meta-accuracy and perspective-taking. The evidence that we found gives us a reason to believe that the link between social competence and meta-accuracy of first impressions on the Internet exists and could be captured better with more specialized measures of Internet social competence.

Meta-accuracy for Internet expressive means varied with proficiency in these means in the case of the text. This implies that meta-accuracy of first impressions does depend on the specificities of the communication environment (Internet). In face-to-face communication, the text is typically presented in the auditory domain rather than the visual domain, where grammar and spelling are less important than written text. Thus, experience in self-expression through written text, including on a meta-accuracy level, is less important offline than it is online. Different communication environments require different proficiency levels for the various expressive means.

#### Biases and Target-Perceiver Sex Combinations

Finally, the observed positive warmth bias for selfies and negative competence bias for text suggest metaperceptual distortion for specific combinations of cognitive dimension and Internet-expressive means. Participants believed that they appeared warmer in their selfies and less competent in their text than they did in reality. These biases not only suggest associations between specific expressive means and perceptual dimensions but further emphasize that meta-accuracy may be affected by Internet specificities. The ways in which such specificities may affect the meta-accuracy factor categories (information, traits, targets, and judges: Funder, 1995; Carlson and Elsaadawy, 2021) present rich material for further investigation. For example, it would be interesting to study whether and how meta-accuracy on both dimensions would be influenced in synchronous online settings where feedback and dynamic cues are available much like in face-to-face interactions, but where aspects such as Internet connection stability may cause artifacts and disrupt the natural information flow.

Overall, the effects were small and mostly present in the combination of male targets and female perceivers. As we had larger than the minimum required sample for the statistical tests, it is unlikely that the effect sizes were due to a lack of statistical power. It is more likely that either the effects are indeed small or that our tools and methods did not capture them properly. The presence of some effects gives sufficient reason to study the matter further with different measures. The presence of effects almost exclusively in the male targets-female perceivers pairing also merits further attention. We believe this phenomenon may be attributed to sex-related specificities of Internet behavior. For instance, it may be that in the role of the target, men exercise less control of expression than women and in the role of perceivers, women are more sensitive than men.

### Limitations

By using an asynchronous setting, we eliminated the possibility of participant interaction (a common element in meta-accuracy research; Tsankova and Tair, 2021a). However, this study was among the first to examine meta-accuracy online, and we are confident that future work will overcome the methodological and ethical challenges associated with synchronous settings. Furthermore, we focused on the target, but future studies also need to examine perceiver-related aspects of first impressions’ meta-accuracy on the Internet (Elsaadawy et al., 2021).

## Conclusion

We presented initial evidence for the underlying perceptual dimensions, role of social competence, and biases in the meta-accuracy of first impressions on the Internet. We provide answers but also identify intriguing questions, which we hope will stimulate future work on the topic.

## Data Availability Statement

The raw data supporting the conclusions of this article will be made available by the authors, without undue reservation.

## Ethics Statement

The studies involving human participants were reviewed and approved by the Ethical Committee of the Institute for Population and Human Studies, Bulgarian Academy of Sciences (IPHS-BAS). The patients/participants provided their online informed consent to participate in this study.

## Author Contributions

ETs programmed the study, collected the data, conducted the statistical analyses, and wrote the initial draft. ETa reviewed and contributed to all manuscript drafts. Both authors contributed to the conceptualization of the study and approve the final version of the manuscript.

## Conflict of Interest

The authors declare that the research was conducted in the absence of any commercial or financial relationships that could be construed as a potential conflict of interest.

## Publisher’s Note

All claims expressed in this article are solely those of the authors and do not necessarily represent those of their affiliated organizations, or those of the publisher, the editors and the reviewers. Any product that may be evaluated in this article, or claim that may be made by its manufacturer, is not guaranteed or endorsed by the publisher.

## Footnotes

1
https://osf.io/95ha6

2
www.prolific.co

3
www.soscisurvey.de
