# Supplementary material for: Meta-Accuracy on the Internet: Initial Tests of Underlying Dimensions, Contributing Factors, and Biases
Source: Front Psychol. 2022 Mar 2;13:837931. doi: 10.3389/fpsyg.2022.837931 (PMC8926076; doi:10.3389/fpsyg.2022.837931)
Supplement: Supplementary file 1 [file Data_Sheet_1.docx]

**A Priori Power Analyses** (G*Power, Version 3.1.9.4, Faul et al., 2007; 2009)

**One-tailed correlations**

expected: medium effect of .30 and power of .80

estimated total sample size: 67

**Repeated measures ANOVAs (RM-ANOVAs)**

expected: medium effect of .25 and power of .80

estimated total sample size: 98 (between-factors effects), 34 (within-factors effects), 34 (within-between interaction)

**two-tailed t-tests**

expected: medium effect of .50 and power of .80

estimated total sample size: 34 (paired samples), 128 (independent samples), 34 (one sample)

Faul, F., Erdfelder, E., Buchner, A., & Lang, A.-G. (2009). Statistical power analyses using G*Power 3.1: Tests for correlation and regression analyses. *Behavior Research Methods, 41*, 1149–1160. <https://doi.org/10.3758/BRM.41.4.1149>

Faul, F., Erdfelder, E., Lang, A.-G., & Buchner, A. (2007). G*Power 3: A flexible statistical power analysis program for the social, behavioral, and biomedical sciences. *Behavior Research Methods, 39*, 175–191. <https://doi.org/10.3758/BF03193146>

*Data Screening and Exclusion*

| **Targets** | **Problem** | **Decision** |
| --- | --- | --- |
|  | 18 participants (6 female) failed to provide complete datasets | Excluded |
|  | 2 participants (1 female) reported having uploaded images other than selfies | Excluded |
|  |  | |
|  | ***Manual inspection*** | |
|  | 1 female participant uploaded a video instead of a selfie | Excluded |
|  | 1 female participant uploaded a group photo | Excluded |
|  | 1 female participant uploaded an image of torso instead of face | Excluded |
|  | 4 participants (3 female) uploaded images of non-humans (a flower, two cartoons, and an animal) | Excluded |
|  | 1 male participant uploaded an image of a famous sports person | Excluded |
|  |  |  |
| **Perceivers** | 6 (4 female) participants failed to provide complete datasets | Excluded |
|  | 1 male participant completed the survey twice | Excluded the second dataset |

Selfies including pets, special garments and, in one case, head gear, face masks, and digitally added effects, were left in the data as we believed these were intentional choices on the side of the targets about self-presentation on social media.

In some cases where the participants had uploaded a screenshot of their selfies (which was occasionally necessary to reduce file size) the image included meta-data such as network connection, etc. These parts of the image were digitally cut out before they were presented to the perceivers. Apart from that, images were not processed and were presented in the size in which they were provided to us in order to maintain high ecological validity with social media where images are found in varying sizes and resolutions.

Texts were copied and pasted in Microsoft PowerPoint in Arial font size 20, while keeping all the targets’ formatting, typos, etc. Perceivers were presented with screenshots of the texts from PowerPoint. Texts were unedited with three exceptions: 1) despite our instructions to not include personal information such as names 1 male participant had disclosed their name which we blurred out prior to presenting their text to the perceivers; 2) our data collection software had transcribed into characters an emoji used by 1 female participant, so we displayed that as an actual emoji to our perceivers; and 3) some information for 1 female participant was changed to two replacement characters (��) by our data collection software and we removed those before showing the respective text to the perceivers. Despite instructions, many targets had entered birthplaces and age. We believed that removing those would have created considerable data loss, and as this information was not deemed sufficient for target identification, we left it in.

**Definitions of Warmth and Competence**

When we say that someone is **warm**, we refer to qualities related to that person’s *intent* (for example, *friendliness*, *helpfulness*, *sincerity*, *trustworthiness*, *morality*, etc.).

When we say that someone is **competent**, we refer to qualities related to that person’s *ability* (for example, *intelligence*, *skill*, *creativity*, *efficacy*, etc.).

based on

Fiske, S. T., Cuddy, A. J. C., & Glick, P. (2007). Universal dimensions of social cognition: warmth and competence. *Trends in Cognitive Sciences, 11*(2), 77–83. <https://doi.org/10.1016/j.tics.2006.11.005>

**The Internet Expressivity Proficiency Questionnaire (IEPQ)**

**Instructions**

Please indicate to what extent each of the following statements about Internet communication applies to you.

The term “images” refers to all kinds of images you may be using on the Internet (photos, which may be selfies or photos in general, as well as avatars, etc.).

**IEPQ-Self**

In the following, please focus on *the way you personally see* your Internet communication.

| *does not apply to me at all* |  |  |  |  |  | *completely applies to me* |
| --- | --- | --- | --- | --- | --- | --- |
| 1 | 2 | 3 | 4 | 5 | 6 | 7 |

| **01. I express myself successfully** in Internet communication. | 1 | 2 | 3 | 4 | 5 | 6 | 7 |
| --- | --- | --- | --- | --- | --- | --- | --- |
| **02. I express myself successfully** through **text** in Internet communication. | 1 | 2 | 3 | 4 | 5 | 6 | 7 |
| **03. I express myself successfully** through **images** in Internet communication. | 1 | 2 | 3 | 4 | 5 | 6 | 7 |
| **04. I express myself successfully** through **emoji/emoticons** in Internet communication. | 1 | 2 | 3 | 4 | 5 | 6 | 7 |
| **05. I express myself successfully** through **video** in Internet communication. | 1 | 2 | 3 | 4 | 5 | 6 | 7 |
| **06. I convey my *feelings* successfully** in Internet communication. | 1 | 2 | 3 | 4 | 5 | 6 | 7 |
| **07. I convey my** ***feelings* successfully** through **text** in Internet communication. | 1 | 2 | 3 | 4 | 5 | 6 | 7 |
| **08. I convey my** ***feelings* successfully** through **images** in Internet communication. | 1 | 2 | 3 | 4 | 5 | 6 | 7 |
| **09. I convey my** ***feelings* successfully** through **emoji/emoticons** in Internet communication. | 1 | 2 | 3 | 4 | 5 | 6 | 7 |
| **10. I convey my** ***feelings* successfully** through **video** in Internet communication. | 1 | 2 | 3 | 4 | 5 | 6 | 7 |
| **11. I convey my** ***thoughts* successfully** in Internet communication. | 1 | 2 | 3 | 4 | 5 | 6 | 7 |
| **12. I convey my** ***thoughts* successfully** through **text** in Internet communication. | 1 | 2 | 3 | 4 | 5 | 6 | 7 |
| **13. I convey my** ***thoughts* successfully** through **images** in Internet communication. | 1 | 2 | 3 | 4 | 5 | 6 | 7 |
| **14. I convey my** ***thoughts* successfully** through **emoji/emoticons** in Internet communication. | 1 | 2 | 3 | 4 | 5 | 6 | 7 |
| **15. I convey my** ***thoughts* successfully** through **video** in Internet communication. | 1 | 2 | 3 | 4 | 5 | 6 | 7 |

**IEPQ-Meta**

In the following, please focus on *the way you believe others see* your Internet communication.

| *does not apply to me at all* |  |  |  |  |  | *completely applies to me* |
| --- | --- | --- | --- | --- | --- | --- |
| 1 | 2 | 3 | 4 | 5 | 6 | 7 |

| **01. Others find that I express myself successfully** in Internet communication. | 1 | 2 | 3 | 4 | 5 | 6 | 7 |
| --- | --- | --- | --- | --- | --- | --- | --- |
| **02. Others find that I express myself successfully** through **text** in Internet communication. | 1 | 2 | 3 | 4 | 5 | 6 | 7 |
| **03. Others find that I express myself successfully** through **images** in Internet communication. | 1 | 2 | 3 | 4 | 5 | 6 | 7 |
| **04. Others find that I express myself successfully** through **emoji/emoticons** in Internet communication. | 1 | 2 | 3 | 4 | 5 | 6 | 7 |
| **05. Others find that I express myself successfully** through **video** in Internet communication. | 1 | 2 | 3 | 4 | 5 | 6 | 7 |
| **06. Others find that I convey my *feelings* successfully** in Internet communication. | 1 | 2 | 3 | 4 | 5 | 6 | 7 |
| **07. Others find that I convey my *feelings* successfully** in Internet communication **where I use text**. | 1 | 2 | 3 | 4 | 5 | 6 | 7 |
| **08. Others find that I convey my *feelings* successfully** in Internet communication **where I use images**. | 1 | 2 | 3 | 4 | 5 | 6 | 7 |
| **09. Others find that I convey my *feelings* successfully** in Internet communication **where I use emoji/emoticons**. | 1 | 2 | 3 | 4 | 5 | 6 | 7 |
| **10. Others find that I convey my *feelings* successfully** in Internet communication **where I use video**. | 1 | 2 | 3 | 4 | 5 | 6 | 7 |
| **11. Others find that I convey my *thoughts* successfully** in Internet communication. | 1 | 2 | 3 | 4 | 5 | 6 | 7 |
| **12. Others find that I convey my *thoughts* successfully** in Internet communication **where I use text**. | 1 | 2 | 3 | 4 | 5 | 6 | 7 |
| **13. Others find that I convey my *thoughts* successfully** in Internet communication **where I use images**. | 1 | 2 | 3 | 4 | 5 | 6 | 7 |
| **14. Others find that I convey my *thoughts* successfully** in Internet communication **where I use emoji/emoticons**. | 1 | 2 | 3 | 4 | 5 | 6 | 7 |
| **15. Others find that I convey my *thoughts* successfully** in Internet communication **where I use video**. | 1 | 2 | 3 | 4 | 5 | 6 | 7 |

**Calculation of IEPQ Total and Subscores**

**IEPQ-Self**

IEPQ-Self General = (IEPQ-Self 01 + IEPQ-Self 02 + IEPQ-Self 03 + IEPQ-Self 04 + IEPQ-Self 05)/5

IEPQ-Self Feelings = (IEPQ-Self 06 + IEPQ-Self 07 + IEPQ-Self 08 + IEPQ-Self 09 + IEPQ-Self 10)/5

IEPQ-Self Thoughts = (IEPQ-Self 11 + IEPQ-Self 12 + IEPQ-Self 13 + IEPQ-Self 14 + IEPQ-Self 15)/5

IEPQ-Self Total = (IEPQ-Self 01 + IEPQ-Self 02 + IEPQ-Self 03 + IEPQ-Self 04 + IEPQ-Self 05 + IEPQ-Self 06 + IEPQ-Self 07 + IEPQ-Self 08 + IEPQ-Self 09 + IEPQ-Self 10 + IEPQ-Self 11 + IEPQ-Self 12 + IEPQ-Self 13 + IEPQ-Self 14 + IEPQ-Self 15)/15

**IEPQ-Meta**

IEPQ-Meta General = (IEPQ-Meta 01 + IEPQ-Meta 02 + IEPQ-Meta 03 + IEPQ-Meta 04 + IEPQ-Meta 05)/5

IEPQ-Meta Feelings = (IEPQ-Meta 06 + IEPQ-Meta 07 + IEPQ-Meta 08 + IEPQ-Meta 09 + IEPQ-Meta 10)/5

IEPQ-Meta Thoughts = (IEPQ-Meta 11 + IEPQ-Meta 12 + IEPQ-Meta 13 + IEPQ-Meta 14 + IEPQ-Meta 15)/5

IEPQ-Meta Total = (IEPQ-Meta 01 + IEPQ-Meta 02 + IEPQ-Meta 03 + IEPQ-Meta 04 + IEPQ-Meta 05 + IEPQ-Meta 06 + IEPQ-Meta 07 + IEPQ-Meta 08 + IEPQ-Meta 09 + IEPQ-Meta 10 + IEPQ-Meta 11 + IEPQ-Meta 12 + IEPQ-Meta 13 + IEPQ-Meta 14 + IEPQ-Meta 15)/15

*Distribution of Targets and Perceivers*

| **Targets** | ***n* Perceivers** | |
| --- | --- | --- |
|  | **Female** | **Male** |
| f01–f05, m01–m05 | 11 | 9 |
| f06–f10, m06–m10 | 10 | 10 |
| f11–f15, m11–m15 | 9 | 11 |
| f16–f20, m16–m20 | 9 | 10 |
| f21–f25, m21–m25 | 10 | 10 |
| f26–f30, m26–m30 | 10 | 10 |
| f31–f35, m31–m35 | 11 | 10 |
| f36–f40, m36–m40 | 10 | 11 |
| f41–f45, m41–m45 | 11 | 10 |
| f46–f50, m46–m50 | 11 | 10 |
| f51–f55, m51–m55 | 10 | 10 |
| f56–f60, m56–m60 | 11 | 10 |
| f61–f65, m61–m65 | 9 | 10 |
| f66–f70, m66–m70 | 10 | 10 |
| f71–f75, m71–m75 | 10 | 11 |
| f76–f80, m76–m80 | 10 | 8 |
| f81–f85, m81–m85 | 9 | 9 |
| f86–f90, m86–m90 | 10 | 11 |
| f91–f03, m91–m95 | 11 | 10 |

*Note.* f = female, m = male; target number determined by survey completion order

**H3 – Preregistration Analysis**

We checked whether meta-accuracy varied with varying proficiency in Internet expressive means in a series of repeated measures analyses of variance (RM-ANOVAs), one for each combination of social dimension, IEPQ version (Self, Meta), and relevant IEPQ subscale (General, Feelings, Thoughts). As two between-subject factors we entered Image Proficiency (high, low) and Text Proficiency (high, low). Both these variables were created based on median splits for the respective questions from each IEPQ subscale. As a within-subject factor we entered Expressive Means (text, images). The text and selfie Meta-Accuracy Indices for both dimensions were dependent variables. We applied Bonferroni correction for multiple comparisons to the post hoc tests.

For the warmth dimension we observed an interaction between Expressive Means and Text Proficiency for the IEPQ-Self-and-Meta General subscales, as well as a tendency for this interaction for the IEPQ-Self Feelings subscale. In particular, IEPQ-Self General: F(1, 94) = 4.90, p = .029, ηp2 = .05, with selfie meta-accuracy (M = 1.38, SD = 0.99) lower than text meta-accuracy (M = 0.91, SD = 0.74), t(59) = 3.28, p = .009, when experience text proficiency was high; IEPQ-Meta General: F(1, 104) = 5.43, p = .022, ηp2 = .05, with selfie meta-accuracy (M = 1.36, SD = 0.80) lower than text meta-accuracy (M = 0.93, SD = 0.82), t(51) = 2.91, p = .027, when text proficiency was high; IEPQ-Self Feelings: F(1, 100) = 3.96, p = .045, ηp2 = .04, with non-significant post hoc comparisons (ps ≥ .1). The IEPQ-Self General results need to be regarded with caution as the equality of variances assumption was not met for selfie Meta-Accuracy Index (Levene’s test F(3, 94) = 5.59, p = .001). Finally, there was also tendency for an interaction between Expressive Means and Image Proficiency for the IEPQ-Meta Thoughts subscale (F(1, 105) = 3.70, p = .057, ηp2 = .03), but with non-significant post hoc comparisons (ps > 1).

For the competence dimension there was a tendency for a main effect of Expressive Means for the IEPQ-Self General subscale: F(1, 94) = 3.54, p = .063, ηp2 = .04, with selfie meta-accuracy (M = 0.96, SD = 0.78) higher than text meta-accuracy (M = 1.18, SD = 0.91). There was also a tendency for a main effect of Image Proficiency for the IEPQ-Self Thoughts subscale (F(1, 108) = 3.46, p = .065, ηp2 = .03), with meta-accuracy higher for low (M = 0.99, SD = 0.77) than for high (M = 1.27, SD = 0.83) image proficiency.

Overall, Internet expressive means proficiency mostly appeared to affect meta-accuracy along the warmth dimension and in the case of text. When experience with text was high, warmth meta-accuracy was higher for texts as compared to selfies.

**Further Exploratory Analyses – Meta-Accuracy Differences**

**Sex**

We computed a series of two-tailed paired samples t-tests comparing meta-accuracy for female and male perceivers separately for the female and male targets. A significant difference was present only in the case of the male targets where text meta-accuracy was higher for female perceivers (*M* = 0.94, *SD* = 0.75) than for male perceivers (*M* = 1.09, *SD* = 0.80), Student’s *t*(95) = -2.65, *p* = .010, *d* = -0.27.

A note of caution: Meta-accuracy here is a composite variable, calculated from both target and perceiver scores. Conceptually, it relates more to the targets than the perceivers and it tells us how strongly targets’ impressions differ from perceivers’ impressions. Meta-accuracy cannot be attributed to the perceivers. The above analysis is exploratory only and it’s goal is to test for sex differences as far as possible, to check for the uniqueness of the male targets-female perceivers combination.

**Social Competence**

Using the TEIQue-SF and IEPQ median splits we calculated in testing H3 we compared the meta-accuracy of cases with high and low target meta-accuracy though a series of two-tailed independent samples t-tests.

For Emotional Intelligence an effect was present only on the TEIQue-SF Self-control subscale where selfie warmth meta-accuracy was higher for targets with low self-control (*M* = 1.03, *SD* = 0.85) compared to targets with high self-control (*M* = 1.29, *SD* = 0.88), Student’s *t*(174) = -1.99, *p* = .049, *d* = -0.30.

In the case of Internet expressivity significant differences emerged again for selfie warmth in the metaperception of general and feelings expression proficiency on the Internet. For general expressivity proficiency, Student’s *t*(181) = -2.30, *p* = .023, *d* = -0.34, with meta-accuracy of low proficiency targets (*M* = 0.97, *SD* = 0.81) higher than that of high proficiency targets (*M* = 1.26, *SD* = 0.89). For feelings expressivity proficiency, Student’s *t*(183) = -2.39, *p* = .018, *d* = -0.35, with meta-accuracy in cases with low proficiency targets (*M* = 0.98, *SD* = 0.82) once more being higher than in cases with high proficiency targets (*M* = 1.28, *SD* = 0.89).

**Social Dimensions**

Two-tailed paired samples t-tests compared meta-accuracy for the warmth and competence dimensions. No difference was present on the level of the complete sample (*p*s >.1). As by now we had observed that some effects were only present for certain target-perceiver sex pairings, we performed the tests also for all four combinations of target-perceiver sex. The tests revealed a tendency for a difference in text meta-accuracy between the dimensions in the male targets-female perceivers pairing, Student’s *t*(95) = -1.95, *p* = .054, *d* = -0.20, with meta-accuracy higher for the warmth (*M* = 0.94, *SD* = 0.75) than for the competence dimension (*M* = 1.15, *SD* = 0.84).

**Expressive Means**

Two-tailed paired samples t-tests did not reveal differences in meta-accuracy for both types of Internet expressive means on the level of the entire sample (*p*s > .1). However, splitting the sample into the four possible target-perceiver sex combinations indicated a difference between the expressive means along the warmth dimension again in the male targets and female perceivers , *t*(95) = 2.56, *p* = .012, *d* = 0.26, where warmth meta-accuracy was lower for selfies (*M* = 1.24, *SD* = 0.93) than for text (*M* = 0.94, *SD* = 0.75).

**Further Exploratory Analyses – Checks**

Our analyses were based on the assumption that our two social competence measures (TEIQue-SF and IEPQ) were positively correlated with each other. To confirm this assumption we computed one-tailed (for positive direction) Pearson correlations on the level of the entire sample (*N* = 189). Indeed, both the Self and Meta versions of the IEPQ were significantly and positively correlated with the well-established TEIQue-SF (*r*_Self_ = .20, p = .003; *r*_Meta_ = .19, p = .004). We attribute the small effect size to the novelty of the IEPQ.

Finally, we conducted two-tailed independent samples t-tests comparing meta-accuracy indices based on median splits for the control questions (self-and-meta-attributed importance of both dimensions to self-presentation and impression formation on the Internet, as well as integration of online and offline self). Significant differences emerged only in the case of self-perceived importance of self-presentation and only for selfies along the *warmth* dimension. There meta-accuracy in cases where targets attributed low importance to online self-presentation (*M* = 0.90, *SD* = 0.85) was higher than meta-accuracy for cases where targets attributed high importance to online self-presentation (*M* = 1.22, *SD* = 0.88), Student’s *t*(149) = -2.21, *p* = .029, *d* = -0.36. The effect is small but could suggest that self-consciousness and overthinking might impair meta-accuracy for selfie warmth.

*Pearson Correlations Between Meta-Accuracy Index and TEIQue-SF Question 2 (Seeing Things from Another’s Perspective)*

|  | Meta-Accuracy Index | | | | | | | | | | |
| --- | --- | --- | --- | --- | --- | --- | --- | --- | --- | --- | --- |
|  |  | | | | | | | | | | |
|  | Warmth | | | | | | | | | | |
|  | Selfie | | | | |  | Text | | | | |
|  | All | FF | FM | MF | MM |  | All | FF | FM | MF | MM |
| TEIQue-SF Q2r | .09 | .02 | .01 | .12 | .13 |  | .01 | .08 | .02 | -.02 | -.05 |
|  |  | | | | | | | | | | |
|  | Competence | | | | | | | | | | |
|  | Selfie | | | | |  | Text | | | | |
|  | All | FF | FM | MF | MM |  | All | FF | FM | MF | MM |
| TEIQue-SF Q2r | -.15^*^ | -.02 | -.10 | -.26^**^ | -.15 |  | -.02 | -.12 | 0 | .05 | .06 |

*Note.* All = all targets, all perceivers; FF = female targets, female perceivers; FM = female targets, male perceivers; MF = male targets, female perceivers; MM = male targets, male perceivers. *N*_All_ = 189, *n*_FF_ = 93, *n*_FM_ = 93, *n*_MF_ = 96, *n*_MM_ = 96.

^*^ *p* < .05, ^**^ *p* < .01, one-tailed for negative correlation (as ability to see things from another’s perspective increases, indicated by the reversed scores in the originally negatively phrased TEIQue-SF Question 2, meta-accuracy should increase, as indicated by smaller difference between targets’ metaperception and others’ perception of them or a decreasing meta-accuracy index).
